# Impact of lymph node dissection on overall survival and cancer-specific survival in elderly patients with early-stage non-small cell lung cancer: a SEER database analysis

**DOI:** 10.57264/cer-2025-0038

**Published:** 2025-12-09

**Authors:** Dong-Chu Zhang, Jiang-Shun Yang, Cun-Qing Zheng

**Affiliations:** 1 Department of Clinical Laboratory, WenZhou Seventh People’s Hospital, Wenzhou 325006, Zhejiang, China; 2 Key Laboratory of Neuropsychiatric Endocrinology, WenZhou Seventh People’s Hospital, Wenzhou 325006, Zhejiang, China

**Keywords:** elderly patients, lymph node dissection, non-small-cell lung cancer, propensity score matching

## Abstract

**Aim:**

The aim of the study is to understand the impact of lymph node dissection (LND) on overall survival (OS) and cancer-specific survival (CSS) in elderly patients with early-stage non-small cell lung cancer (NSCLC), and to find the best characteristics of the beneficiary population.

**Materials & methods:**

Based on the Surveillance, Epidemiology, and End Results (SEER) database, the effect of LND on OS and CSS in elderly patients with early-stage NSCLC was analyzed using a retrospective method. Multivariate Cox regression model was employed to determine the factors influencing OS and CSS in elderly patients with early-stage NSCLC. Kaplan–Meier and sequential landmark analyses were conducted to estimate and compare the survival curves and median follow-up time of patients.

**Results:**

The study included 27,540 participants. The age distribution of patients who underwent LND was mostly 65–74 years old (61.8%). 83.2% received chemotherapy concurrently, and 9.0% received radiotherapy. A total of 10,240 patients were successfully matched after propensity score matching. Elderly patients with early-stage NSCLC who received LND (OS_median_: 60 vs 23; CSS_median_: 136 vs 32) had significantly improved OS and CSS, with consistent results from sequential landmark analysis of long-term survivors. The results of subgroup analyses displayed that factors such as gender, age, marital status, grade and tumor size affected the prognosis of elderly patients with early-stage NSCLC who received LND.

**Conclusion:**

The OS and CSS in patients with early-stage elderly NSCLC who underwent LND at different time points after diagnosis were significantly improved.

## Plain language summary

**What is this article about?:**

In this paper, early-stage non-small cell lung cancer patients were analyzed to explore the prognosis of patients after lymph node dissection (LND) and the factors that influence it.

**What were the results?:**

There is a positive impact on overall survival and cancer-specific survival from LND in non-small cell lung cancer patients at various time points after diagnosis, and specific patient subgroups may benefit more from LND.

**What do the results mean?:**

Proper selection of LND candidates based on patient characteristics can help improve quality of life and reduce mortality.

In recent years, the global burden of lung cancer has continued to increase. According to 2022 global cancer statistics, lung cancer accounted for 2.48 million new cases worldwide, representing 12.4% of all malignancies and ranking as the leading cause of cancer-related deaths [1]. Non-small cell lung cancer (NSCLC), the predominant pathological subtype accounting for approximately 85% of all lung cancer cases, has emerged as a major public health challenge in cancer prevention and control [2]. Notably, NSCLC incidence shows a pronounced age concentration, with the highest prevalence occurring between 60 and 70 years old and a relatively low proportion of patients under 50. As global population aging accelerates, the number of elderly NSCLC patients is projected to continue rising, making personalized treatment strategies for older patients particularly urgent [3].

In clinical practice, significant differences exist between elderly and younger NSCLC patients in terms of treatment response and prognosis. Some studies have revealed differences in postoperative complications and mortality between older and younger lung cancer patients, with a higher recurrence rate in older patients. Particularly in advanced cases with distant metastasis, treatment outcomes and prognosis are significantly poorer [4–6]. However, with increased awareness of medical screening and diagnostic techniques, the detection rate of early-stage (operable) lung cancer has increased from 25% to approximately 63%, and continues to show an upward trend [7,8], creating favorable conditions for implementing curative treatment. The treatment of early-stage cancers can significantly improve patient survival. Against this backdrop, developing scientifically sound and effective surgical strategies for early-stage NSCLC patients-particularly the elderly-has become a key focus of current clinical research.

Currently, treatment options for early-stage NSCLC include surgery, radiotherapy, chemotherapy and laser ablation [9]. Surgical resection provides optimal local control and offers a survival advantage over chemotherapy and radiotherapy alone for suitable surgical candidates [10]. Lymph node dissection (LND) is a commonly performed surgical procedure aimed at removing the extent of lymph node metastasis in the region surrounding the tumor [11]. However, the actual benefits and risks of LND remain somewhat controversial and require further investigation. Yang *et al.* suggested that LND could potentially benefit non-small cell patients with lung cancer [12], while a single-center retrospective study by Wu *et al.* did not report a significant survival advantage associated with LND, but rather suggested a potential increase in the risk of surgical complications and mortality [13]. Demographic characteristics and clinicopathological documents of patients with early-stage lung cancer showed significant differences, resulting in varying prognoses for patients with different characteristics [14]. Therefore, identifying the subgroup of patients who truly benefit holds significant clinical importance.

The characteristics of the elderly NSCLC population most likely to benefit from LND and the factors that may influence the postoperative outcome of LND remain unclear. Therefore, this study utilized the Surveillance, Epidemiology, and End Results (SEER) database to investigate the impact of LND on overall survival (OS) and cancer-specific survival (CSS) in early-stage NSCLC patients over 65 years old. It further analyzes potential factors affecting postoperative outcomes to identify the optimal beneficiary population, thereby providing evidence-based medical support for individualized surgical strategies in this cohort.

## Materials & methods

### Patient selection & study design

The clinical database ‘SEER program’ collects cancer incidence, prevalence and survival data from the US Cancer Registry, covering approximately 28% of the US population from 18 states and representing all regions of the country [15]. This study was a retrospective cohort analysis. In this population-based study, the elderly NSCLC patient data were downloaded from the SEER*Stat Database: Incidence – SEER Research Data, 17 Registries, Nov 2023 Sub (2000–2021). SEER*Stat version 8.4.4 (https://seer.cancer.gov/data-software/) was used to obtain the patient information.

**Figure 1.**
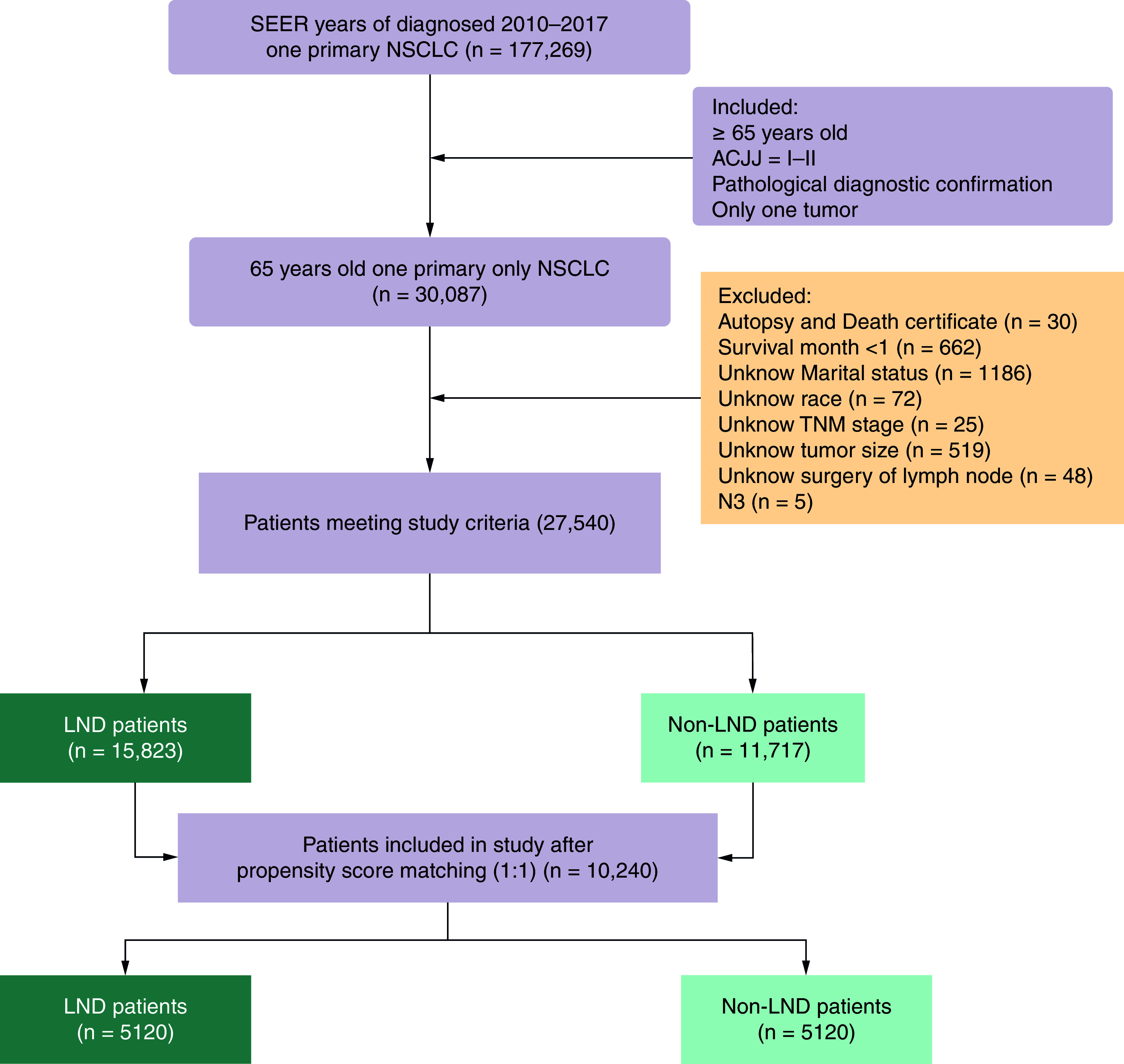
Patient selection process based on inclusion and exclusion criteria. AJCC: American Joint Committee on Cancer; LND: Lymph node dissection; NSCLC: Non-small cell lung cancer; SEER: Surveillance, Epidemiology, and End Results.

Elderly patients diagnosed with primary early-stage NSCLC between 2010 and 2017 were selected as study subjects. The follow-up period commenced from the date of NSCLC diagnosis and concluded upon death or capping at 31 December 2021, whichever occurred first. Data from patients who were alive, lost to follow-up or died from other causes at the final follow-up were treated as censored data (coded as 0 in the status variable). The specific inclusion criteria were as follows: year of diagnosis was 2010–2017; ≥65 years old; stage I–II; only one primary tumor; diagnosed with NSCLC; diagnostic criteria were microscopic pathological examination. Exclusion criteria: the source of cases was autopsy and death certificate; survival time <1 month; unknown information about demographic characteristics (marital status, race); unknown clinical information (stage, tumor node metastasis stage [staging according to the seventh edition of the American Joint Committee on Cancer (seventh AJCC)], tumor size and grade); and unknown treatment (LND, chemotherapy and radiotherapy). We excluded metastasis in contralateral mediastinal, contralateral hilar, ipsilateral or contralateral scalene or supraclavicular lymph node(s) (N3) cases because of the small number, which may make the match fail. Figure 1 displayed the inclusion process for our participants.

### Definition of variables

The following histologic codes were used to define NSCLC [16]: 8010, 8012:8014, 8020:8022, 8050:8052, 8070:8078, 8140:8147, 8255, 8260, 8310, 8323, 8480, 8481, 8490, 8550, 8572.

OS was defined as the length of time between cancer diagnosis and death of any cause. CSS was defined as the length of time between cancer diagnosis and death due to NSCLC.

Marital status was categorized as other and married. Based on median and quartiles 2.6 (1.8, 4.0), the variable of tumor size was classified into four groups: <1.8, 1.8–2.5, 2.6–4 and 4.1+ cm.

### Data analyses

Frequencies and percentages were employed to describe categorical data. Intergroup comparisons were conducted using the chi-square test or Fisher’s exact test. For LND usage differences, propensity score matching (PSM) was employed to achieve 1:1 matching via a logistic regression model. The PSM utilized the nearest neighbor matching method with a caliper value of 0.05. The matching variables included: age, gender, race, marital status, tumor size, the extent of the primary tumor (T), lymph node (N) involvement, grade, radiation and chemotherapy.

Univariate analysis was performed using unadjusted Cox regression. Multivariate Cox regression model was utilized to explore the factors influencing the prognosis of elderly patients with early-stage NSCLC, and the variable inclusion method was stepwise regression backward. Given the multiple hypothesis testing performed, we also applied Holm’s method to correct the p-values for multiple factors. Propensity scores were incorporated into pre-matching Cox regression models for sensitivity analysis. Survival curves and median follow-up time of patients were estimated and compared using Kaplan–Meier analysis.

Because the treatment time of LND was unknown, the time of enrolment was different. We performed sequential landmark analysis of the effect of LND on patient OS and CSS for patients surviving ≥ 1 or ≥2 years after diagnosis, to eliminate the treatment time bias [17,18]. Subgroup analysis was performed on some differing variables after matching.

All statistical analyses in this study were conducted using R software (4.4.1), with statistical significance set at p < 0.05.

## Results

### Baseline characteristics

According to the inclusion and exclusion criteria, this study encompassed 27,540 elderly patients with early-stage NSCLC, with 71.1% in stage I and 28.9% in stage II. The age distribution of patients was mostly concentrated between 65 and 84 years old, with fewer patients aged 85 years or older (8.7%). Racial distribution was primarily white (84.3%). There was little difference in the percentage of males and females, with 51.6% males and 48.4% females. For tumor characteristics, there were more patients with T1 (47.5%), T2 (40.2%), N0 (89.6%), grade II (36.9%) and grade III (27.9%). Most patients underwent chemotherapy (83.0%), while 31.7% received radiotherapy and 57.5% had LND. Among LND recipients, 61.8% were aged 65–74 years, 83.2% of the patients received concurrent chemotherapy and 9.0% underwent radiotherapy (Table 1).

**Table 1.** Patients' characteristic information before and after propensity score matching in lymph node dissection and nonlymph node dissection groups.

Variables	Before PSM				After PSM			
	Total n (%)	Non-LND n (%)	LND n (%)	p-value	Total n (%)	Non-LND n (%)	LND n (%)	p-value
**All patients**	27,540	11,717	15823		10,240	5120	5120	
**Age (years)**								
65–74	14,433 (52.4)	4655 (39.7)	9778 (61.8)	<0.001	5201 (50.8)	2506 (48.9)	2695 (52.6)	<0.001
75–84	10,709 (38.9)	5211 (44.5)	5498 (34.7)		4081 (39.9)	2155 (42.1)	1926 (37.6)	
85+	2398 (8.7)	1851 (15.8)	547 (3.5)		958 (9.4)	459 (9.0)	499 (9.7)	
**Gender**								
Male	13,317 (48.4)	5685 (48.5)	7632 (48.2)	0.648	4930 (48.1)	2555 (49.9)	2375 (46.4)	<0.001
Female	14,223 (51.6)	6032 (51.5)	8191 (51.8)		5310 (51.9)	2565 (50.1)	2745 (53.6)	
**Race**								
White	23,220 (84.3)	9783 (83.5)	13437 (84.9)	<0.001	8185 (79.9)	4308 (84.1)	3877 (75.7)	<0.001
Black	2173 (7.9)	1117 (9.5)	1056 (6.7)		1053 (10.3)	443 (8.7)	610 (11.9)	
Other	2147 (7.8)	817 (7.0)	1330 (8.4)		1002 (9.8)	369 (7.2)	633 (12.4)	
**Marital status**								
Married	14,502 (52.7)	5355 (45.7)	9147 (57.8)	<0.001	5079 (49.6)	2539 (49.6)	2540 (49.6)	1.000
Other	13,038 (47.3)	6362 (54.3)	6676 (42.2)		5161 (50.4)	2581 (50.4)	2580 (50.4)	
**Chemotherapy**								
None/unknown	22,858 (83.0)	9694 (82.7)	13164 (83.2)	0.322	7853 (76.7)	4057 (79.2)	3796 (74.1)	<0.001
Yes	4682 (17.0)	2023 (17.3)	2659 (16.8)		2387 (23.3)	1063 (20.8)	1324 (25.9)	
**Radiation**								
None/unknown	18,807 (68.3)	4409 (37.6)	14398 (91.0)	<0.001	7457 (72.8)	3747 (73.2)	3710 (72.5)	0.424
Yes	8733 (31.7)	7308 (62.4)	1425 (9.0)		2783 (27.2)	1373 (26.8)	1410 (27.5)	
**Tumor size(cm)**								
<1.8	6148 (22.3)	2359 (20.1)	3789 (23.9)	<0.001	2185 (21.3)	1081 (21.1)	1104 (21.6)	0.036
1.8–2.5	7310 (26.5)	2795 (23.9)	4515 (28.5)		2289 (22.4)	1205 (23.5)	1084 (21.2)	
2.6–4.0	7802 (28.3)	3449 (29.4)	4353 (27.5)		2867 (28.0)	1418 (27.7)	1449 (28.3)	
4.1+	6280 (22.8)	3114 (26.6)	3166 (20.0)		2899 (28.3)	1416 (27.7)	1483 (29.0)	
**N**								
N0	24,666 (89.6)	10,756 (91.8)	13,910 (87.9)	<0.001	8909 (87.0)	4564 (89.1)	4345 (84.9)	<0.001
N1	2825 (10.3)	953 (8.1)	1872 (11.8)		1305 (12.7)	551 (10.8)	754 (14.7)	
N2	49 (0.2)	8 (0.1)	41 (0.3)		26 (0.3)	5 (0.1)	21 (0.4)	
**T**								
T1	13,085 (47.5)	5540 (47.3)	7545 (47.7)	<0.001	4054 (39.6)	2215 (43.3)	1839 (35.9)	<0.001
T2	11,076 (40.2)	4384 (37.4)	6692 (42.3)		4477 (43.7)	2077 (40.6)	2400 (46.9)	
T3	3353 (12.2)	1772 (15.1)	1581 (10.0)		1697 (16.6)	821 (16.0)	876 (17.1)	
T4	26 (0.1)	21 (0.2)	5 (0.0)		12 (0.1)	7 (0.1)	5 (0.1)	
**Grade**								
Grade I	3445 (12.5)	1041 (8.9)	2404 (15.2)	<0.001	1076 (10.5)	561 (11.0)	515 (10.1)	<0.001
Grade II	10,157 (36.9)	2913 (24.9)	7244 (45.8)		3394 (33.1)	1728 (33.8)	1666 (32.5)	
Grade III	7689 (27.9)	2932 (25.0)	4757 (30.1)		3195 (31.2)	1523 (29.7)	1672 (32.7)	
Grade IV	242 (0.9)	74 (0.6)	168 (1.1)		152 (1.5)	46 (0.9)	106 (2.1)	
Unknown	6007 (21.8)	4757 (40.6)	1250 (7.9)		2423 (23.7)	1262 (24.6)	1161 (22.7)	

LND: Lymph node dissection; PSM: Propensity score matching.

### Propensity score matching

**Figure 2.**
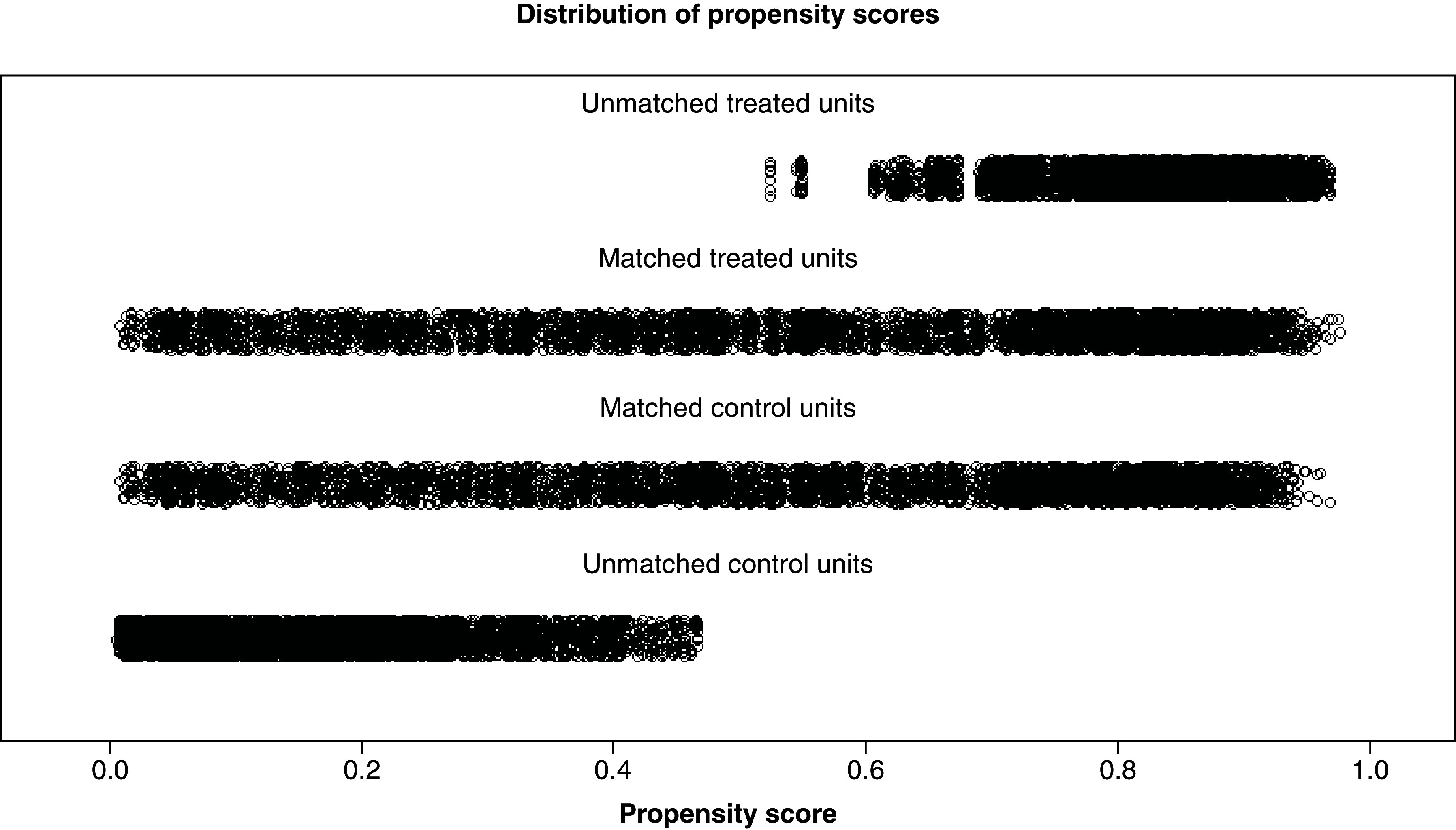
Propensity score matching for lymph node dissection and nonlymph node dissection groups.

Analysis of 10 variables revealed statistically significant differences (p < 0.05) between the LND and non-LND groups, except for gender and chemotherapy. All variables were matched using PSM to control for confounding factors, and a total of 10,240 patients were finally successfully matched between the two groups. Before matching, the LND group showed higher covariate scores than the non-LND group. After matching, the scores of the two groups were more evenly distributed, with some variables showing p > 0.05. The variables remaining differences between the two groups would be analyzed by stratification in subsequent analyses (Table 1 & Figure 2). Additionally, we calculated Nagelkerke’s R^2^ to assess the performance of the logistic regression model used to derive propensity scores. The pre-matching propensity score model demonstrated good explanatory power (Nagelkerke’s R^2^ = 0.501), indicating significant baseline imbalance between the LND and non-LND groups. Following PSM, the explanatory power for the same covariates decreased substantially (Nagelkerke’s R^2^ = 0.045), indicating that the matching procedure successfully balanced measurable covariates between the two groups, with treatment assignment in the matched cohort approximating random distribution. This demonstrates that PSM was highly successful.

### Independent prognostic factors in elderly patients with early-stage NSCLC

In univariate analyses, the 11 variables involving demographic characteristics, tumor clinicopathology and treatment were all associated with OS and CSS in elderly patients with early-stage NSCLC. These variables were subsequently included in multivariate COX regression analyses. Ultimately, all 11 variables included were found to be independent factors affecting OS and CSS in elderly early-stage NSCLC (Tables 2 & 3). After PSM, LND demonstrated a significant positive impact on both OS and CSS (HR_OS_: 0.44, 95% CI: 0.42–0.46; HR*css*: 0.40, 95% CI: 0.38–0.43, all adjusted p Holm <0.001). With increasing T and N stage as well as higher grade, the HR of OS and CSS gradually increased, indicating poorer survival outcomes. Sensitivity analysis results indicate that undergoing LND was also significantly associated with OS and CSS (HR_OS_: 0.41, 95% CI: 0.39–0.43; HR*css*: 0.36, 95% CI: 0.34–0.38, all adjusted p Holm <0.001) (Table 4), validating the robustness of our findings.

**Table 2.** Univariate and multivariate Cox proportional hazards regression analysis of the overall survival of elderly patients with early-stage non-small cell lung cancer.

Variables	Before PSM					After PSM				
	Univariate analysis		Multivariate analysis			Univariate analysis		Multivariate analysis		
	HR (95%CI)	p-value	HR (95%CI)	Original p-value	Adjusted *p*-Holm	HR (95%CI)	p-value	HR (95%CI)	Original p-value	Adjusted *p*-Holm
**Age**										
65–74 years	Reference		Reference			Reference		Reference		
75–84 years	1.52 (1.48–1.57)	<0.001	1.32 (1.27–1.36)	<0.001	<0.001	1.33 (1.27–1.40)	<0.001	1.34 (1.28–1.41)	<0.001	<0.001
≥85 years	2.43 (2.31–2.55)	<0.001	1.60 (1.52–1.69)	<0.001	<0.001	1.61 (1.49–1.74)	<0.001	1.74 (1.60–1.88)	<0.001	<0.001
**Sex**										
Male	Reference		Reference			Reference		Reference		
Female	0.72 (0.70–0.74)	<0.001	0.72 (0.69–0.74)	<0.001	<0.001	0.70 (0.67–0.73)	<0.001	0.71 (0.67–0.74)	<0.001	<0.001
**Race**										
White	Reference		Reference			Reference		Reference		
Black	1.06 (1.01–1.12)	0.021	0.94 (0.89–0.99)	0.025	0.102	0.86 (0.79–0.92)	<0.001	0.96 (0.88–1.04)	0.278	0.500
Other	0.74 (0.69–0.78)	<0.001	0.75 (0.71–0.80)	<0.001	<0.001	0.60 (0.55–0.66)	<0.001	0.73 (0.67–0.80)	<0.001	<0.001
**Marital status**										
Married	Reference		Reference			Reference		Reference		
Other	1.24 (1.20–1.27)	<0.001	1.18 (1.15–1.22)	<0.001	<0.001	1.12 (1.07–1.18)	<0.001	1.20 (1.15–1.26)	<0.001	<0.001
**Chemotherapy**										
None/unknown	Reference		Reference			Reference		Reference		
Yes	1.22 (1.17–1.26)	<0.001	0.81 (0.77–0.85)	<0.001	<0.001	1.13 (1.07–1.19)	<0.001	0.82 (0.77–0.88)	<0.001	<0.001
**Radiation**										
None/unknown	Reference		Reference			Reference		Reference		
Yes	2.07 (2.01–2.14)	<0.001	0.96 (0.93–1.00)	0.065	0.135	1.42 (1.35–1.49)	<0.001	1.13 (1.07–1.19)	<0.001	<0.001
**Tumor size(cm)**										
<1.8	Reference		Reference			Reference		Reference		
1.8–2.5	1.37 (1.30–1.43)	<0.001	1.32 (1.26–1.39)	<0.001	<0.001	1.56 (1.45–1.68)	<0.001	1.47 (1.36–1.58)	<0.001	<0.001
2.6–4.0	1.76 (1.69–1.84)	<0.001	1.48 (1.41–1.56)	<0.001	<0.001	1.73 (1.61–1.86)	<0.001	1.62 (1.49–1.75)	<0.001	<0.001
>4.0	2.57 (2.46–2.69)	<0.001	1.92 (1.81–2.03)	<0.001	<0.001	2.39 (2.23–2.56)	<0.001	2.14 (1.96–2.34)	<0.001	<0.001
**N**										
N0	Reference		Reference			Reference		Reference		
N1	1.40 (1.34–1.47)	<0.001	1.55 (1.48–1.63)	<0.001	<0.001	1.41 (1.32–1.51)	<0.001	1.55 (1.44–1.66)	<0.001	<0.001
N2	1.01 (0.69–1.48)	0.952	1.24 (0.85–1.81)	0.269	0.269	1.29 (0.81–2.05)	0.280	1.75 (1.10–2.78)	0.019	0.075
**T**										
T1	Reference		Reference			Reference		Reference		
T2	1.49 (1.44–1.54)	<0.001	1.15 (1.10–1.20)	<0.001	<0.001	1.40 (1.33–1.47)	<0.001	1.07 (1.00–1.14)	0.051	0.145
T3	2.10 (2.01–2.20)	<0.001	1.64 (1.56–1.73)	<0.001	<0.001	1.70 (1.59–1.82)	<0.001	1.49 (1.37–1.61)	<0.001	<0.001
T4	2.62 (1.69–4.06)	<0.001	1.57 (1.01–2.44)	0.045	0.135	1.66 (0.86–3.19)	0.129	1.19 (0.61–2.29)	0.611	0.622
**Grade**										
Grade I	Reference		Reference			Reference		Reference		
Grade II	1.62 (1.53–1.72)	<0.001	1.53 (1.45–1.62)	<0.001	<0.001	1.60 (1.46–1.75)	<0.001	1.51 (1.37–1.65)	<0.001	<0.001
Grade III	2.25 (2.12–2.38)	<0.001	1.76 (1.66–1.87)	<0.001	<0.001	1.92 (1.75–2.10)	<0.001	1.74 (1.58–1.90)	<0.001	<0.001
Grade IV	2.17 (1.85–2.54)	<0.001	1.80 (1.53–2.11)	<0.001	<0.001	1.80 (1.47–2.20)	<0.001	2.01 (1.64–2.45)	<0.001	<0.001
Unknown	2.95 (2.78–3.12)	<0.001	1.63 (1.53–1.73)	<0.001	<0.001	1.76 (1.60–1.93)	<0.001	1.66 (1.51–1.83)	<0.001	<0.001
**LND**										
No	Reference		Reference			Reference		Reference		
Yes	0.36 (0.35–0.38)	<0.001	0.39 (0.37–0.41)	<0.001	<0.001	0.48 (0.46–0.50)	<0.001	0.44 (0.42–0.46)	<0.001	<0.001

CI: Confidence interval; HR: Hazard ratio; LND: Lymph node dissection; PSM: Propensity score matching.

**Table 3.** Univariate and multivariate Cox proportional hazards regression analysis of the cancer-specific survival of elderly patients with early-stage non-small cell lung cancer.

Variables	Before PSM					After PSM				
	Univariate analysis		Multivariate analysis			Univariate analysis		Multivariate analysis		
	HR (95%CI)	p-value	HR (95%CI)	Original p-value	Adjusted p-Holm	HR (95%CI)	p-value	HR (95%CI)	Original p-value	Adjusted p-Holm
**Age**										
65–74 years	Reference		Reference			Reference		Reference		
75–84 years	1.42 (1.37–1.48)	<0.001	1.23 (1.18–1.28)	<0.001	<0.001	1.24 (1.17–1.31)	<0.001	1.27 (1.19–1.35)	<0.001	<0.001
≥85 years	2.22 (2.08–2.36)	<0.001	1.45 (1.36–1.55)	<0.001	<0.001	1.34 (1.21–1.48)	<0.001	1.49 (1.35–1.65)	<0.001	<0.001
**Sex**										
Male	Reference		Reference			Reference		Reference		
Female	0.72 (0.70–0.75)	<0.001	0.75 (0.72–0.78)	<0.001	<0.001	0.70 (0.66–0.74)	<0.001	0.74 (0.69–0.78)	<0.001	<0.001
**Race**										
White	Reference		Reference			Reference		Reference		
Black	1.09 (1.02–1.17)	0.014)	0.94 (0.88–1.00)	0.064	0.117	0.83 (0.75–0.91)	<0.001	0.92 (0.83–1.01)	0.093	0.251
Other	0.80 (0.75–0.86)	<0.001	0.82 (0.76–0.88)	<0.001	<0.001	0.65 (0.58–0.72)	<0.001	0.79 (0.71–0.88)	<0.001	<0.001
**Marital status**										
Married	Reference		Reference			Reference		Reference		
Other	1.22 (1.17–1.27)	<0.001	1.16 (1.11–1.21)	<0.001	<0.001	1.09 (1.03–1.16)	0.002	1.17 (1.10–1.24)	<0.001	<0.001
**Chemotherapy**										
None/unknown	Reference		Reference			Reference		Reference		
Yes	1.60 (1.53–1.67)	<0.001	0.92 (0.87–0.97)	0.001	0.004	1.37 (1.29–1.46)	<0.001	0.89 (0.83–0.96)	0.003	0.010
**Radiation**										
None/unknown	Reference		Reference			Reference		Reference		
Yes	1.98 (1.91–2.06)	<0.001	0.83 (0.79–0.87)	<0.001	<0.001	1.42 (1.34–1.51)	<0.001	1.03 (0.96–1.10)	0.381	0.551
**Tumor size (cm)**										
<1.8	Reference		Reference			Reference		Reference		
1.8–2.5	1.54 (1.45–1.65)	<0.001	1.47 (1.37–1.57)	<0.001	<0.001	1.82 (1.65–2.01)	<0.001	1.67 (1.51–1.84)	<0.001	<0.001
2.6–4.0	2.27 (2.14–2.42)	<0.001	1.75 (1.63–1.87)	<0.001	<0.001	2.17 (1.97–2.38)	<0.001	1.91 (1.72–2.12)	<0.001	<0.001
>4.0	3.86 (3.63–4.10)	<0.001	2.42 (2.25–2.61)	<0.001	<0.001	3.32 (3.03–3.64)	<0.001	2.68 (2.39–3.00)	<0.001	<0.001
**N**										
N0	Reference		Reference			Reference		Reference		
N1	1.76 (1.67–1.85)	<0.001	1.83 (1.72–1.94)	<0.001	<0.001	1.67 (1.54–1.80)	<0.001	1.79 (1.64–1.94)	<0.001	<0.001
N2	1.32 (0.87–2.00)	0.199	1.50 (0.99–2.28)	0.059	0.117	1.90 (1.20–3.02)	0.007	2.38 (1.49–3.79)	<0.001	0.002
**T**										
T1	Reference		Reference			Reference		Reference		
T2	1.93 (1.85–2.01)	<0.001	1.28 (1.21–1.36)	<0.001	<0.001	1.68 (1.57–1.79)	<0.001	1.15 (1.06–1.25)	<0.001	0.005
T3	3.01 (2.85–3.18)	<0.001	2.08 (1.94–2.22)	<0.001	<0.001	2.21 (2.04–2.39)	<0.001	1.78 (1.62–1.96)	<0.001	<0.001
T4	4.45 (2.84–6.99)	<0.001	2.28 (1.44–3.58)	<0.001	0.002	2.38 (1.19–4.76)	0.015	1.48 (0.73–2.98)	0.273	0.551
**Grade**										
Grade I	Reference		Reference			Reference		Reference		
Grade II	1.80 (1.66–1.94)	<0.001	1.60 (1.48–1.74)	<0.001	<0.001	1.74 (1.55–1.96)	<0.001	1.57 (1.39–1.76)	<0.001	<0.001
Grade III	2.70 (2.50–2.92)	<0.001	1.93 (1.78–2.09)	<0.001	<0.001	2.19 (1.95–2.47)	<0.001	1.84 (1.64–2.08)	<0.001	<0.001
Grade IV	2.70 (2.21–3.29)	<0.001	1.99 (1.63–2.43)	<0.001	<0.001	2.10 (1.64–2.69)	<0.001	2.24 (1.75–2.87)	<0.001	<0.001
Unknown	3.44 (3.18–3.72)	<0.001	1.80 (1.65–1.95)	<0.001	<0.001	2.04 (1.80–2.30)	<0.001	1.83 (1.62–2.07)	<0.001	<0.001
**LND**										
No	Reference		Reference			Reference		Reference		
Yes	0.35 (0.33–0.36)	<0.001	0.34 (0.32–0.35)	<0.001	<0.001	0.45 (0.43–0.48)	<0.001	0.40 (0.38–0.43)	<0.001	<0.001

CI: Confidence interval; HR: Hazard ratio; LND: Lymph node dissection; PSM: Propensity score matching.

**Table 4.** The results of sensitivity analysis.

Variables	OS			CSS		
	HR (95%CI)	Original p-value	Adjusted p-Holm	HR (95%CI)	Original p-value	Adjusted p-Holm
**Age**						
65–74 years	Reference			Reference		
75–84 years	1.14 (1.09–1.18)	<0.001	<0.001	1.02 (0.97–1.07)	0.496	0.660
≥85 years	1.10 (1.01–1.19)	0.030	0.060	0.88 (0.79–0.98)	0.022	0.108
**Sex**						
Male	Reference			Reference		
Female	0.73 (0.71–0.75)	<0.001	<0.001	0.77 (0.74–0.80)	<0.001	<0.001
**Race**						
White	Reference			Reference		
Black	0.86 (0.81–0.91)	<0.001	<0.001	0.83 (0.78–0.90)	<0.001	<0.001
Other	0.74 (0.70–0.79)	<0.001	<0.001	0.80 (0.74–0.86)	<0.001	<0.001
**Marital status**						
Married	Reference			Reference		
Other	1.07 (1.04–1.11)	<0.001	<0.001	1.02 (0.98–1.07)	0.325	0.660
**Chemotherapy**						
None/unknown	Reference			Reference		
Yes	0.89 (0.85–0.93)	<0.001	<0.001	1.04 (0.98–1.10)	0.201	0.660
**Radiation**						
None/unknown	Reference			Reference		
Yes	0.44 (0.39–0.51)	<0.001	<0.001	0.31 (0.26–0.36)	<0.001	<0.001
**Tumor size(cm)**						
<1.8	Reference			Reference		
1.8–2.5	1.38 (1.32–1.45)	<0.001	<0.001	1.55 (1.45–1.66)	<0.001	<0.001
2.6–4.0	1.46 (1.38–1.53)	<0.001	<0.001	1.71 (1.59–1.83)	<0.001	<0.001
>4.0	1.75 (1.65–1.86)	<0.001	<0.001	2.15 (1.99–2.33)	<0.001	<0.001
**N**						
N0	Reference			Reference		
N1	1.71 (1.63–1.81)	<0.001	<0.001	2.07 (1.95–2.21)	<0.001	<0.001
N2	1.64 (1.12–2.40)	0.011	0.044	2.16 (1.41–3.29)	<0.001	0.003
**T**						
T1	Reference			Reference		
T2	1.24 (1.19–1.30)	<0.001	<0.001	1.42 (1.34–1.51)	<0.001	<0.001
T3	1.63 (1.55–1.72)	<0.001	<0.001	2.06 (1.93–2.20)	<0.001	<0.001
T4	1.36 (0.87–2.11)	0.173	0.173	1.89 (1.20–2.98)	0.006	0.037
**Grade**						
Grade I	Reference			Reference		
Grade II	1.57 (1.48–1.66)	<0.001	<0.001	1.65 (1.53–1.79)	<0.001	<0.001
Grade III	1.68 (1.58–1.78)	<0.001	<0.001	1.81 (1.67–1.96)	<0.001	<0.001
Grade IV	1.78 (1.52–2.09)	<0.001	<0.001	1.97 (1.61–2.40)	<0.001	<0.001
Unknown	1.11 (1.02–1.21)	0.019	0.057	1.09 (0.97–1.22)	0.165	0.660
**LND**						
No	Reference			Reference		
Yes	0.41 (0.39–0.43)	<0.001	<0.001	0.36 (0.34–0.38)	<0.001	<0.001
**Propensity score**	0.19 (0.15–0.25)	<0.001	<0.001	0.12 (0.09–0.17)	<0.001	<0.001

CSS: Cancer-specific survival; CI: Confidence interval; HR: Hazard ratio; LND: Lymph node dissection; OS: Overall survival.

### Impact of LND on survival in elderly patients with early-stage NSCLC

**Figure 3.**
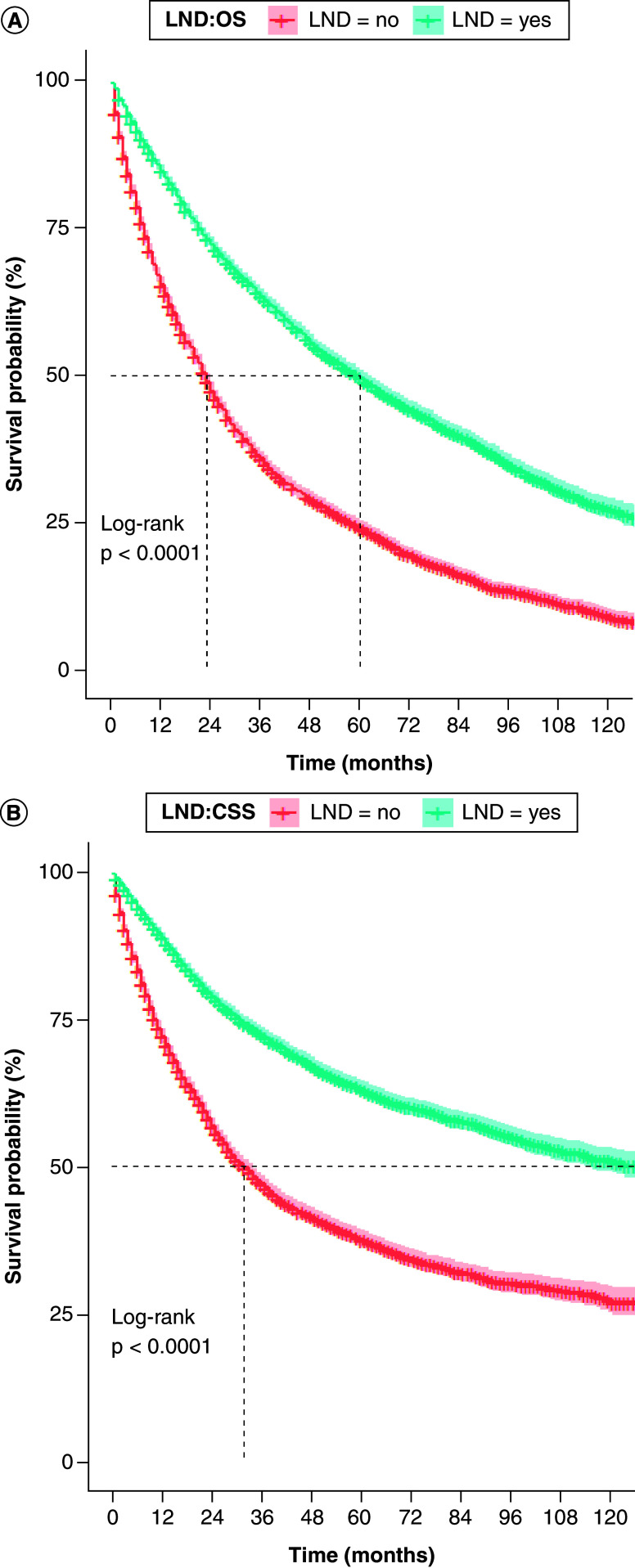
Kaplan–Meier survival curves. **(A)** OS and **(B)** CSS in LND and non-LND groups. CSS: Cancer-specific survival; LND: Lymph node dissection; OS: Overall survival.

Kaplan–Meier analysis revealed that the median OS was 60 months in the LND group and 23 months in the Non-LND group; the 5-year and 10-year OS rates were 49.5% and 27.1% in the LND group, respectively, compared with 23.8% and 8.7% in the Non-LND group (p < 0.001) (Figure 3A). Median CSS was 136 months in the LND group and 32 months in the Non-LND group. The 5-year and 10-year CSS rates were 63.2% and 51.1% in the LND group, versus 37.4% and 27.0% in the Non-LND group, respectively (p < 0.001) (Figure 3B).

### Comparison of survival curves among subgroups between LND & non-LND

**Figure 4.**
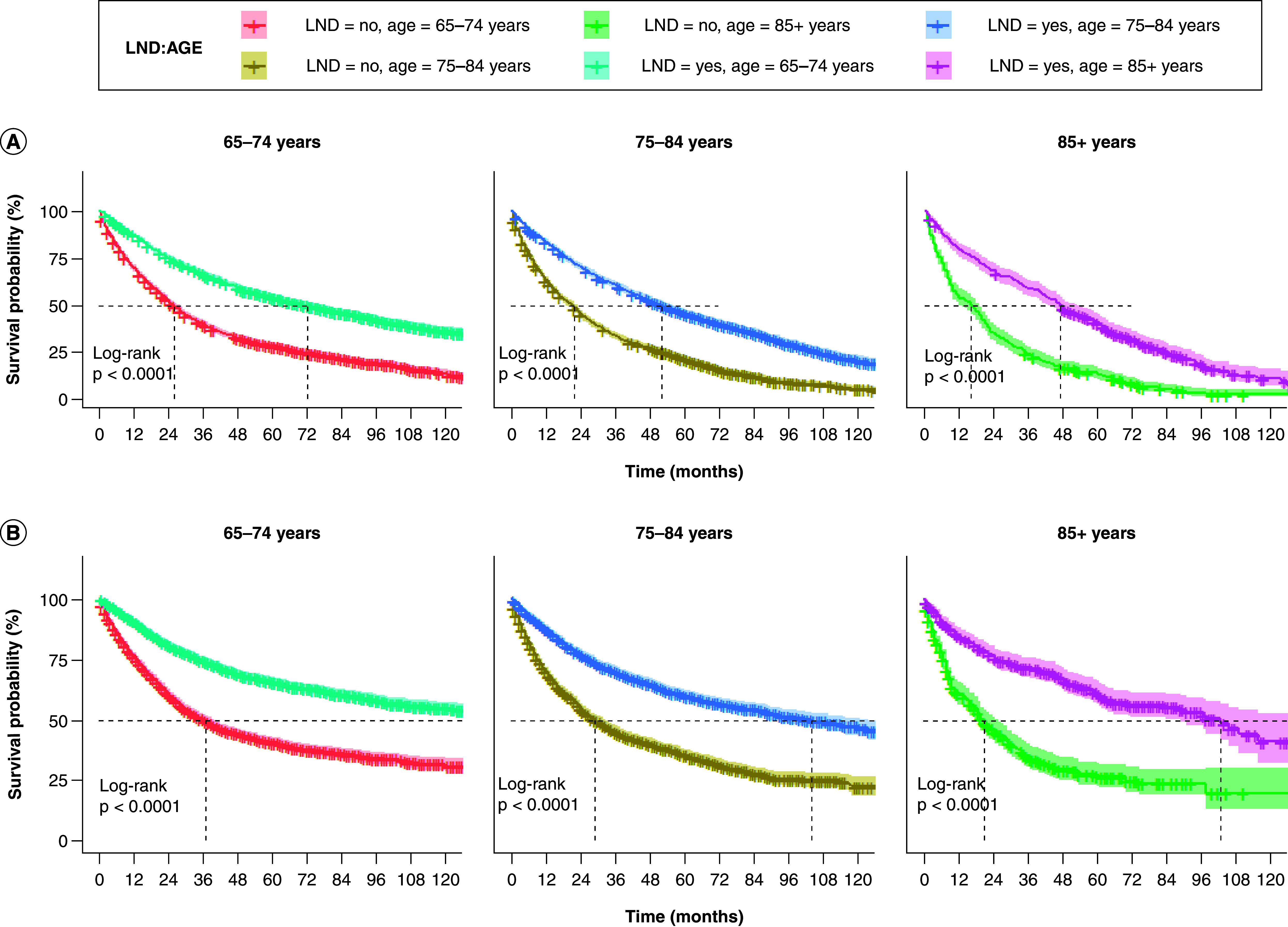
Comparison of overall survival and cancer-specific survival among elderly patients with early-stage non-small cell lung cancer in different age in lymph node dissection and non-lymph node dissection groups. **(A)** OS and **(B)** CSS. CSS: Cancer-specific survival; LND: Lymph node dissection; NSCLC: Non-small cell lung cancer; OS: Overall survival.

**Figure 5.**
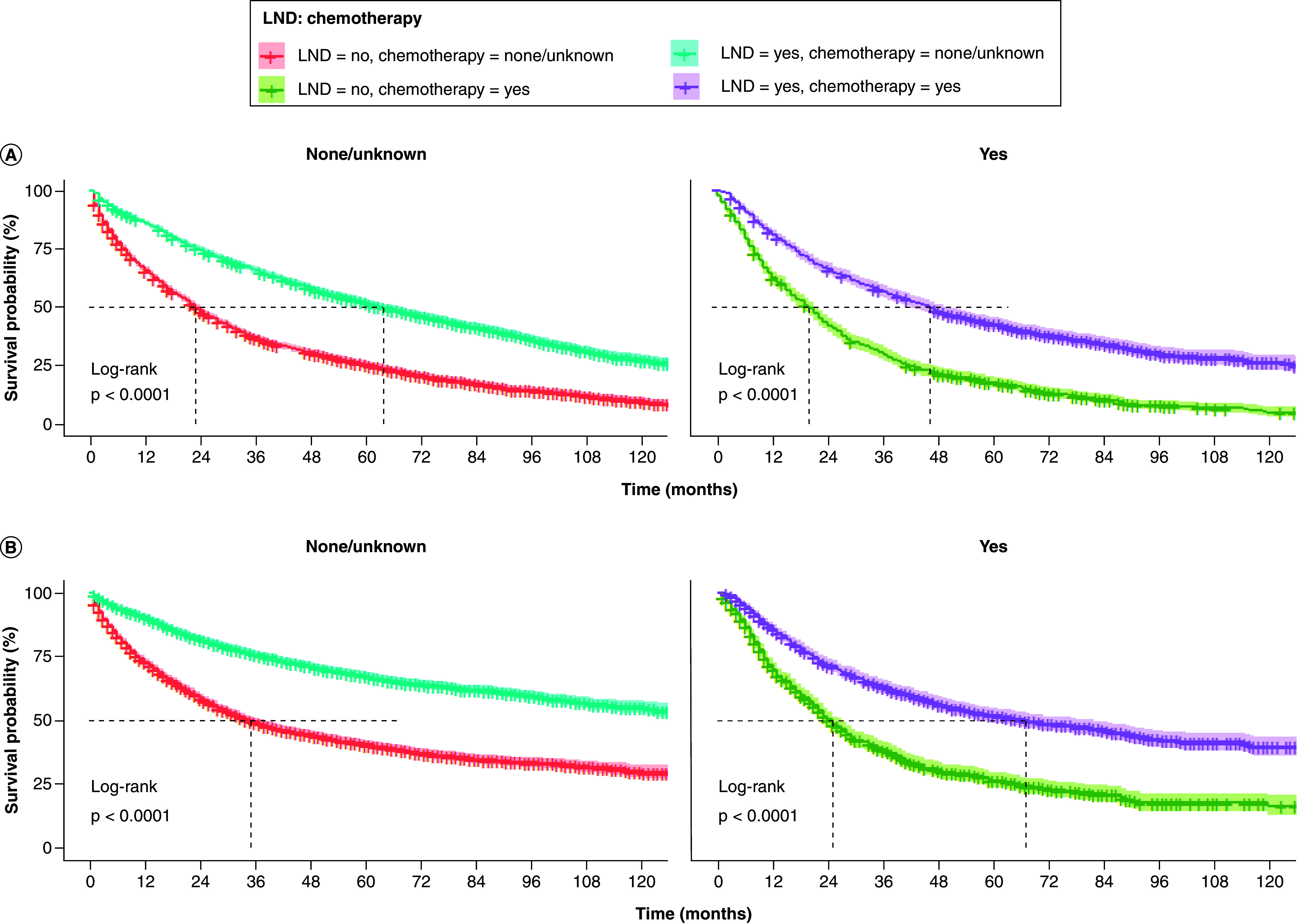
Comparison of overall survival and cancer-specific survival among elderly patients with early-stage non-small cell lung cancer in different chemotherapy in lymph node dissection and non-lymph node dissection groups. **(A)** OS and **(B)** CSS. CSS: Cancer-specific survival; LND: Lymph node dissection; NSCLC: Non-small cell lung cancer; OS: Overall survival.

Due to the statistically significant variables in the results of Cox multivariate analysis and the unbalanced distribution of certain variables between LND and non-LND groups in the matched dataset, subgroup analyses were performed on the matched 10,240 patients. Notably, in all subgroup analyses, LND patients consistently demonstrated better OS and CSS than non-LND patients. There was a decreasing trend in OS and CSS median survival as the degree of tumor-related features increased in the T, N, grade, and tumor size variables (p < 0.001, see Supplementary Figures 1–7). LND improved OS and CSS across all age groups compared with non-LND. However, the degree of improvement decreased with increasing age (Figure 4A & B). OS and CSS median survival were higher in patients receiving chemotherapy combined with LND than those receiving chemotherapy alone (Figure 5A & B).

### Landmark analysis

**Figure 6.**
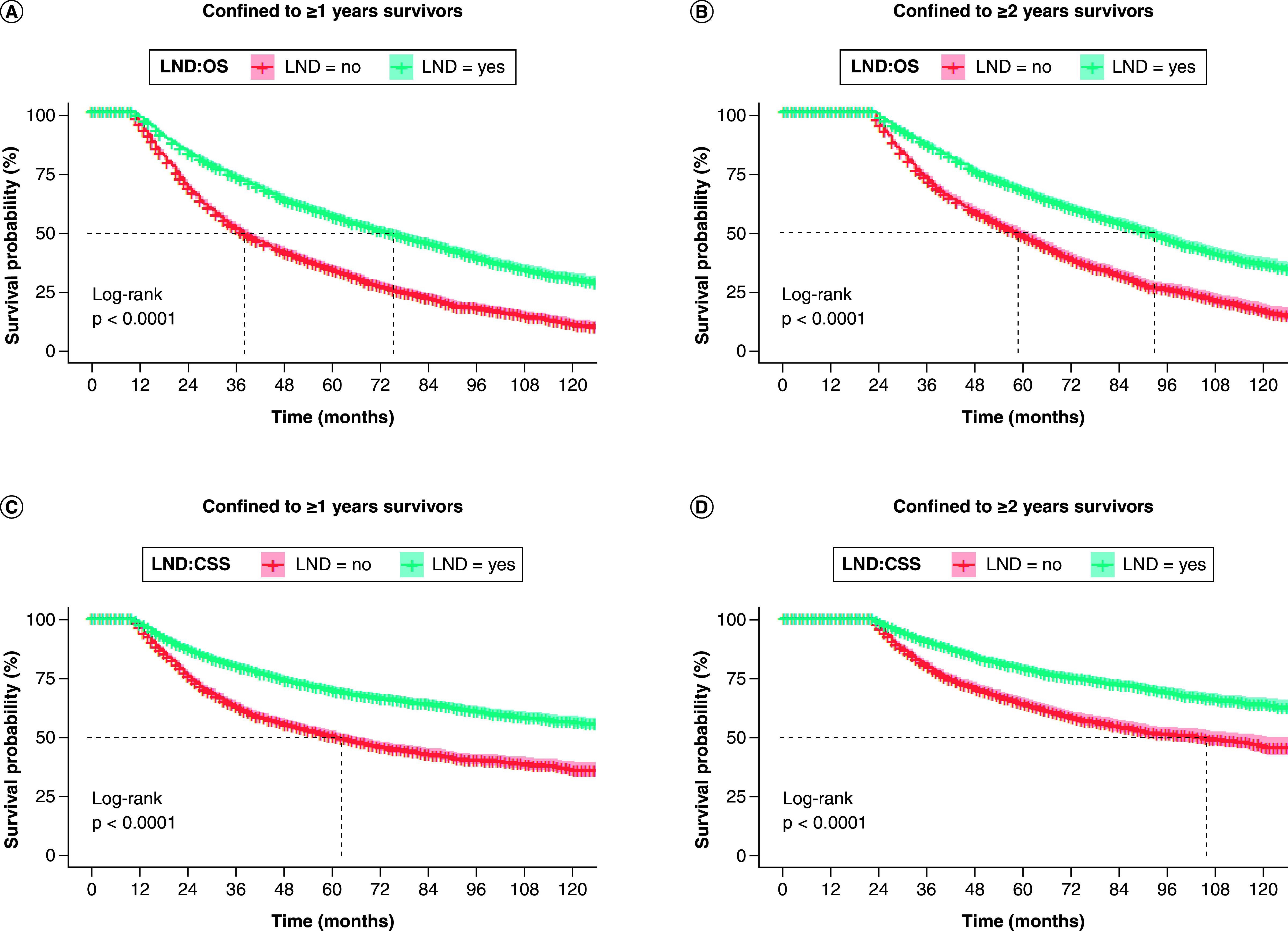
Landmark analyses of overall survival and cancer-specific survival for ≥1 and ≥2 year survivors with early-stage non-small cell lung cancer in lymph node dissection and non-lymph node dissection groups. **(A & B)** OS and **(C & D)** CSS. CSS: Cancer-specific survival; LND: Lymph node dissection; NSCLC: Non-small cell lung cancer; OS: Overall survival.

To eliminate external time bias, we performed a landmark analysis of elderly patients who survived 1 years and 2 years after diagnosis of early-stage NSCLC. The beneficial effect of receiving LND remained in long-term survivors of ≥1 or ≥2 years, and patients in the LND group had significantly higher OS and CS than those in the Non-LND group (Figure 6A & B).

## Discussion

In this study, we investigated the factors influencing the prognosis of elderly patients with early-stage NSCLC and further analyzed which characteristics favoured the acceptance of LND. We identified 11 independent predictors associated with the prognosis of elderly patients with early-stage NSCLC, including demographic characteristics (age, gender, race, marital status), tumor characteristics (tumor size, N-stage, T-stage, grade) and treatment (chemotherapy, radiation, LND), similar to the results of Yu [14].

For patients with early-stage NSCLC, OS and CSS were best in the surgical treatment alone group, with similar results found in most subgroup analyses, and the same results can be obtained in older patients with early-stage NSCLC [9,19]. As LND is a frequently employed surgical method to potentially remove the primary tumor, its impact on elderly early-stage NSCLC patients warranted investigation. The results of this study revealed improved OS and CSS in elderly early-stage NSCLC patients who underwent LND, corroborating the results of Deng and Zhao *et al.* [11,20]. In this analysis, the 5-year OS survival rate was 49.5% and the 5-year CSS survival rate was 63.2% in patients treated with LND, indicating a longer life expectancy compared with those who did not receive LND.

To focus on the differences of OS and CSS between ≥1 and ≥2 year survivors, landmark analysis was used to exclude the bias of treatment time (Figure 4). Notably, at both time points, our data suggest that LND (compared with non-LND) improved OS and CSS in long-term survivors, which is a novel finding. Thus, there is a benefit in OS and CSS for LND in early-stage elderly NSCLC patients at different time points after diagnosis.

In addition, factors such as age, chemotherapy and grade have a significant impact on LND. They also have an impact on the prognosis of patients. Subgroup analyses showed that the LND group demonstrated a significant survival advantage across patient demographic characteristics and clinical information (age, grade, chemotherapy, gender, race, stage, tumor size). Age is typically associated with the prognosis of patients, and the results of this study indicated that patients aged 85 years or older had the poorest OS and CSS (HR_OS_: 1.74, 95% CI: 1.60–1.88; HRcss: 1.49, 95% CI: 1.35–1.65). This may be attributed to declining nutritional status, further reduction in physiological reserves, more complex underlying diseases and decreased treatment tolerance. Furthermore, elderly patients are prone to immune senescence, enabling tumors to bypass immune surveillance. Consequently, primary tumors in elderly NSCLC patients tend to be more aggressive at the time of initial diagnosis [21]. Research indicates that surgical removal is associated with long-term survival outcomes in a significant proportion of patients with early-stage lung cancer in their eighties and beyond [3]. As populations age, cancer burden increases among the elderly [22], so clinical management for elderly patients with early-stage NSCLC should be further improved. Tumor stage and grade in the LND group influenced prognosis, probably because poorly differentiated tumors are considered to be more aggressive, leading to a higher risk of local recurrence and metastasis, which negatively impacts patient survival outcomes [23]. Moreover, subgroup analysis showed that chemotherapy combined with surgery also improved survival outcomes compared with chemotherapy alone, aligning with previous findings [16]. Therefore, these factors should be thoroughly considered when determining treatment to ensure patients receive optimal treatment.

Our findings indicate that LND can benefit elderly patients with early-stage NSCLC, with certain subgroups potentially benefiting more. However, this study has limitations. First, as a retrospective study, there is some selection bias. Second, the SEER database lacks some important information, such as data on inclusion criteria of the population, the type and duration of surgery and other comorbidities. Third, the database was unable to obtain information on some influencing factors, such as smoking history and lifestyle. Although some statistical methods were used to control for suspected confounding variables, the inherent limitations of retrospective studies could not be eliminated. Therefore, prospective studies are necessary to validate these results.

## Conclusion

The prognosis of OS and CSS in elderly patients with early-stage NSCLC is affected by age, gender, race, marital status, tumor size, N-stage, T-stage, grade and treatments, including chemotherapy, radiation and LND. LND may be beneficial for elderly patients with early-stage NSCLC. There is a positive impact on OS and CSS from LND in early-stage elderly NSCLC patients at various time points after diagnosis, and specific patient subgroups may benefit more from LND.

## Summary points

- Non-small cell lung cancer (NSCLC) is the main type of lung cancer.
- Demographic characteristics and clinicopathological documents of patients with early-stage lung cancer were significant differences.
- Different characteristics may result in varying prognoses for patients.
- Using early-stage elderly NSCLC patients as participants.
- Exploring the impact of lymph node dissection (LND) on patients’ prognosis.
- Propensity scores matching controls confounding factors.
- LND improves the prognosis of early-stage elderly NSCLC patients.
- Gender, age, marital status, grade and tumor size affected the prognosis of elderly patients with early-stage NSCLC who received LND.

## Supplementary Material


