# Supplementary material for: Impact of lymph node dissection on overall survival and cancer-specific survival in elderly patients with early-stage non-small cell lung cancer: a SEER database analysis
Source: J Comp Eff Res. 2025 Dec 9;15(1):e250038. doi: 10.57264/cer-2025-0038 (PMC12711093; doi:10.57264/cer-2025-0038)
Supplement: Supplementary file 1 [file cer-15-250038-s1.pdf]

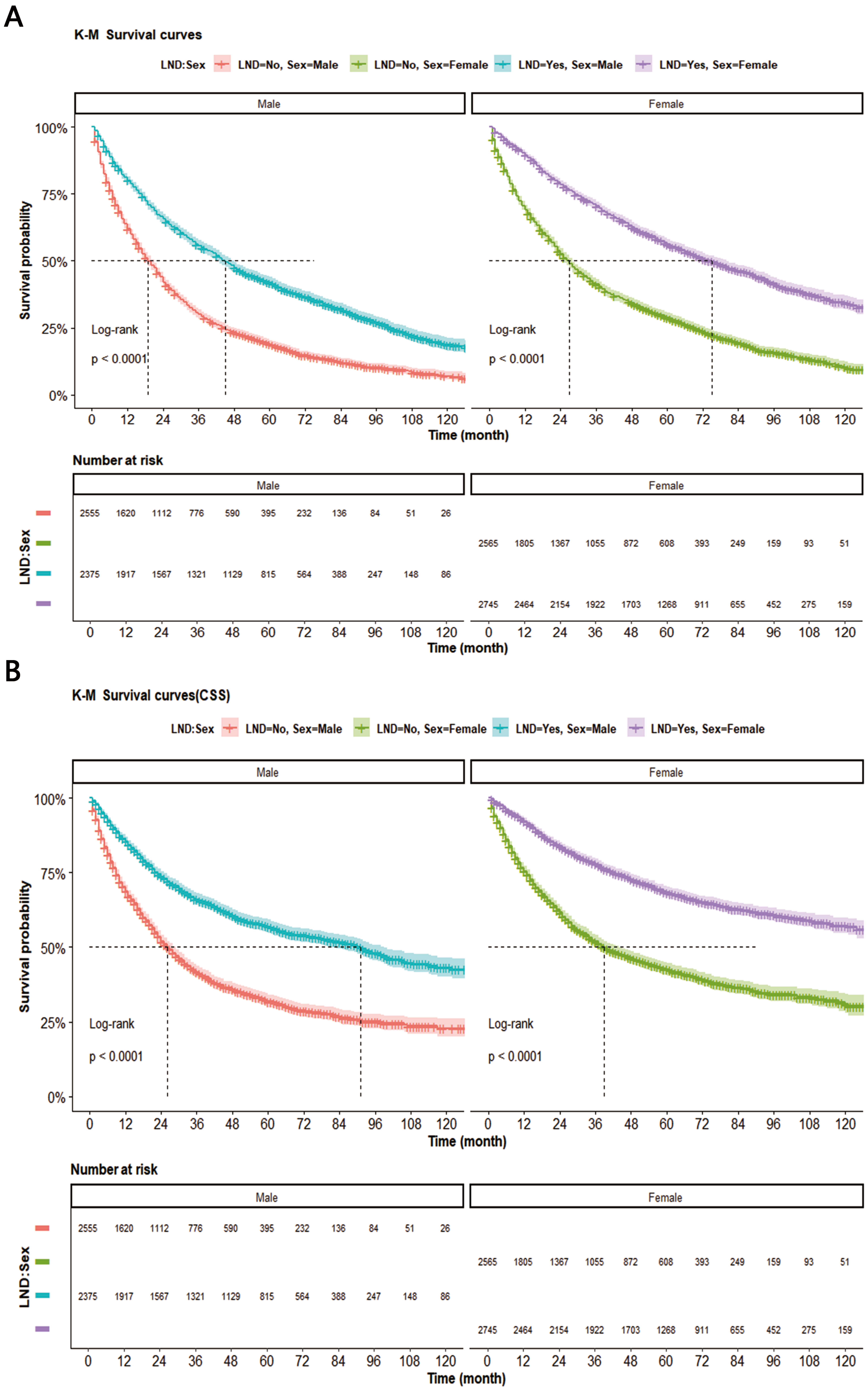
A
K-M Survival curves
LND:Sex
LND=No, Sex=Male
LND=No, Sex=Female
LND=Yes, Sex=Male
LND=Yes, Sex=Female
Male
Female
Survival probability
100%
75%
50%
25%
0%
Log-rank
p < 0.0001
0 12 24 36 48 60 72 84 96 108 120
Time (month)
Number at risk
LND:Sex
2555 1620 1112 776 590 395 232 136 84 51 26
2375 1917 1567 1321 1129 815 564 388 247 148 86
2565 1805 1367 1055 872 608 393 249 159 93 51
2745 2464 2154 1922 1703 1268 911 655 452 275 159
B
K-M Survival curves(CSS)
LND:Sex
LND=No, Sex=Male
LND=No, Sex=Female
LND=Yes, Sex=Male
LND=Yes, Sex=Female
Male
Female
Survival probability
100%
75%
50%
25%
0%
Log-rank
p < 0.0001
0 12 24 36 48 60 72 84 96 108 120
Time (month)
Number at risk
LND:Sex
2555 1620 1112 776 590 395 232 136 84 51 26
2375 1917 1567 1321 1129 815 564 388 247 148 86
2565 1805 1367 1055 872 608 393 249 159 93 51
2745 2464 2154 1922 1703 1268 911 655 452 275 159

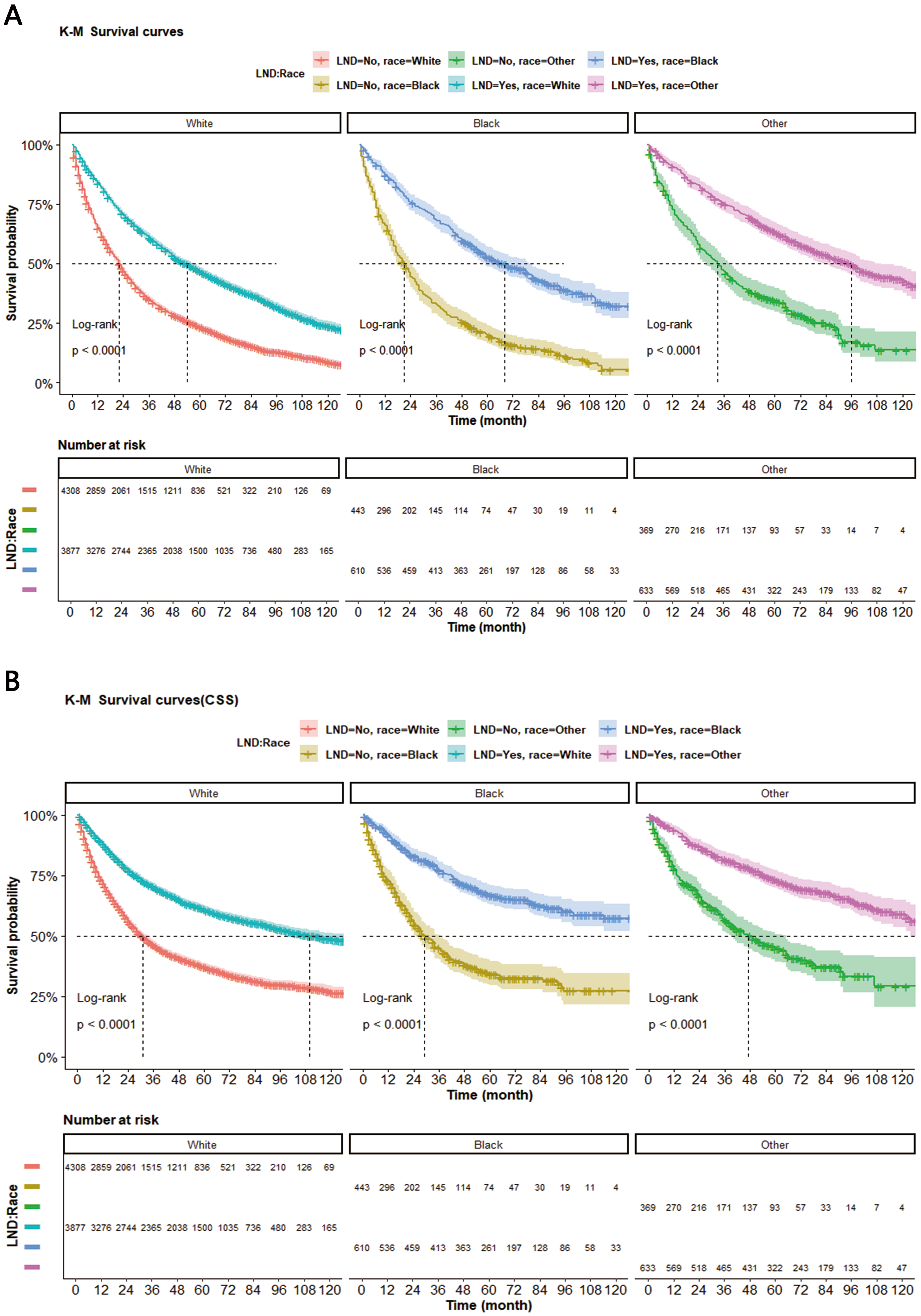
A
K-M Survival curves
LND:Race
LND=No, race=White
LND=No, race=Black
LND=No, race=Other
LND=Yes, race=White
LND=Yes, race=Black
LND=Yes, race=Other
White
Black
Other
Survival probability
100%
75%
50%
25%
0%
Log-rank
p < 0.0001
0 12 24 36 48 60 72 84 96 108 120
Time (month)
Number at risk
4308 2859 2061 1515 1211 836 521 322 210 126 69
3877 3276 2744 2365 2038 1500 1035 736 480 283 165
443 296 202 145 114 74 47 30 19 11 4
610 536 459 413 363 261 197 128 86 58 33
369 270 216 171 137 93 57 33 14 7 4
633 569 518 465 431 322 243 179 133 82 47
B
K-M Survival curves(CSS)
LND:Race
LND=No, race=White
LND=No, race=Black
LND=No, race=Other
LND=Yes, race=White
LND=Yes, race=Black
LND=Yes, race=Other
White
Black
Other
Survival probability
100%
75%
50%
25%
0%
Log-rank
p < 0.0001
0 12 24 36 48 60 72 84 96 108 120
Time (month)
Number at risk
4308 2859 2061 1515 1211 836 521 322 210 126 69
3877 3276 2744 2365 2038 1500 1035 736 480 283 165
443 296 202 145 114 74 47 30 19 11 4
610 536 459 413 363 261 197 128 86 58 33
369 270 216 171 137 93 57 33 14 7 4
633 569 518 465 431 322 243 179 133 82 47

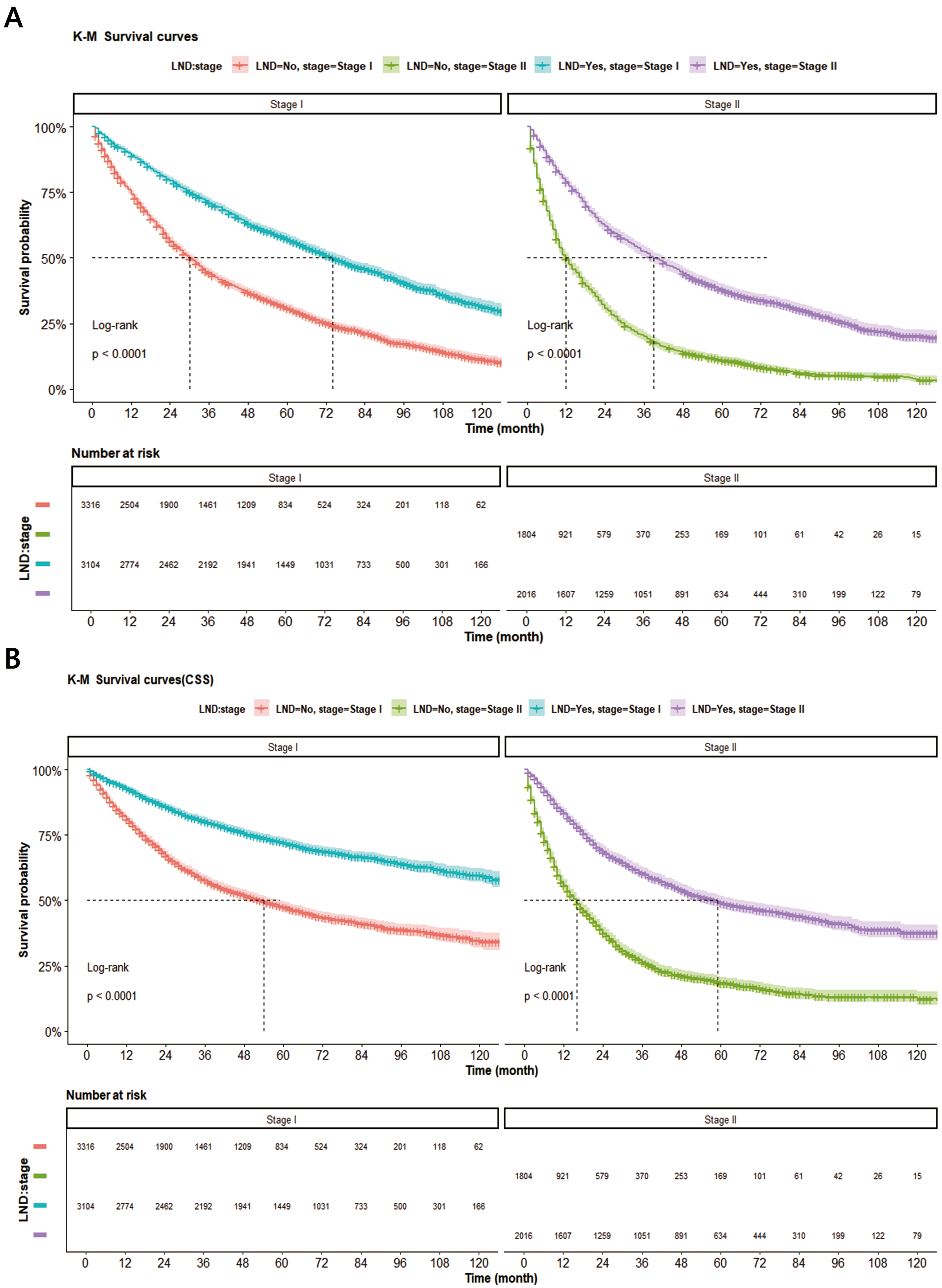
A
K-M Survival curves
LND:stage
LND=No, stage=Stage I
LND=No, stage=Stage II
LND=Yes, stage=Stage I
LND=Yes, stage=Stage II
Stage I
Stage II
Survival probability
100%
75%
50%
25%
0%
Log-rank
p < 0.0001
Time (month)
Number at risk
LND:stage
3316 2504 1900 1461 1209 834 524 324 201 118 62
3104 2774 2462 2192 1941 1449 1031 733 500 301 166
1804 921 579 370 253 169 101 61 42 26 15
2016 1607 1259 1051 891 634 444 310 199 122 79
0 12 24 36 48 60 72 84 96 108 120
B
K-M Survival curves(CSS)
LND:stage
LND=No, stage=Stage I
LND=No, stage=Stage II
LND=Yes, stage=Stage I
LND=Yes, stage=Stage II
Stage I
Stage II
Survival probability
Log-rank
p < 0.0001
Time (month)
Number at risk
LND:stage
3316 2504 1900 1461 1209 834 524 324 201 118 62
3104 2774 2462 2192 1941 1449 1031 733 500 301 166
1804 921 579 370 253 169 101 61 42 26 15
2016 1607 1259 1051 891 634 444 310 199 122 79

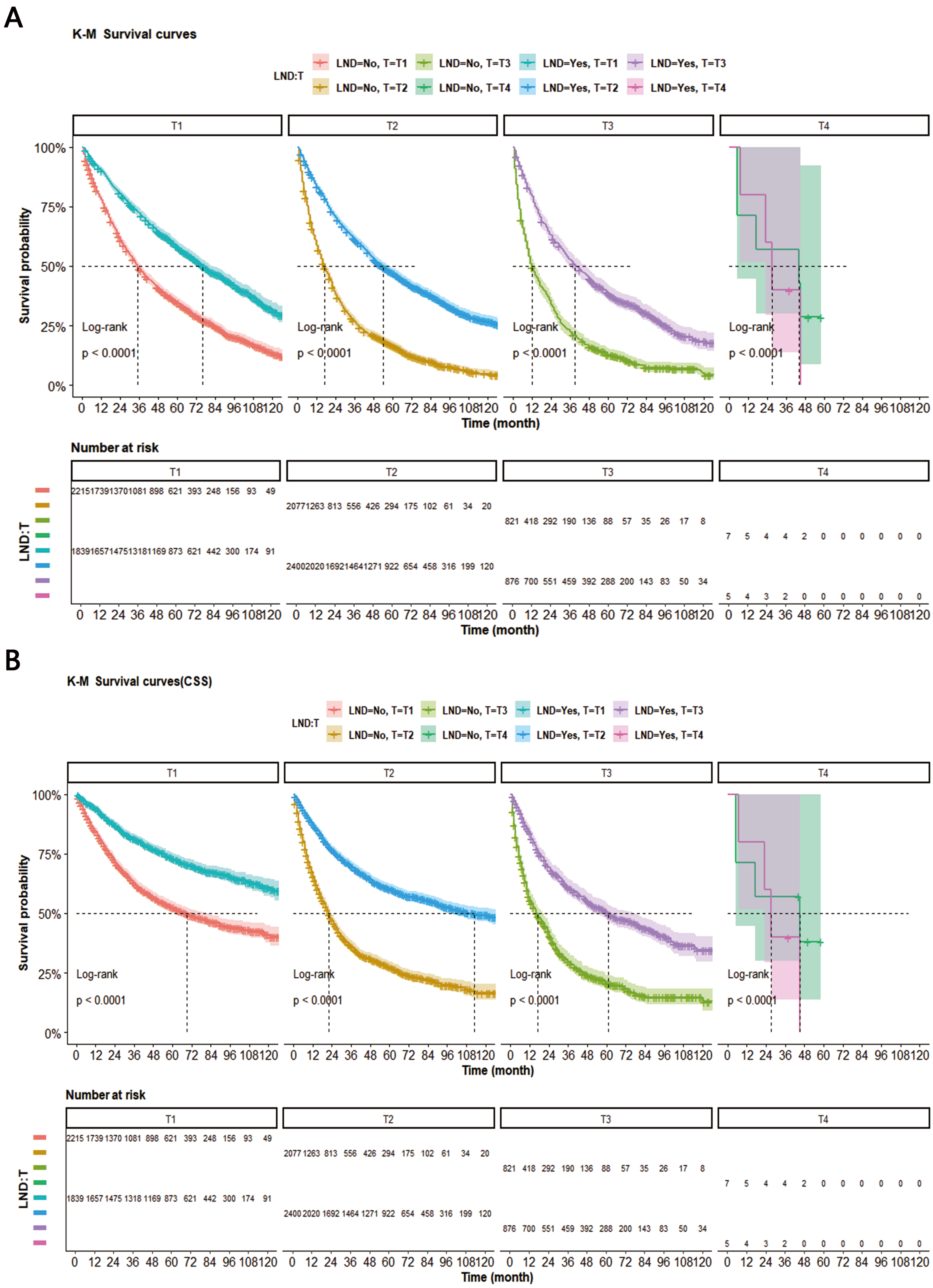
A
K-M Survival curves
LND:T
LND=No, T=T1
LND=No, T=T2
LND=No, T=T3
LND=No, T=T4
LND=Yes, T=T1
LND=Yes, T=T2
LND=Yes, T=T3
LND=Yes, T=T4
T1
T2
T3
T4
Survival probability
100%
75%
50%
25%
0%
Log-rank
p < 0.0001
0 12 24 36 48 60 72 84 96 108 120
Time (month)
Number at risk
LND:T
2215 1739 1370 1081 898 621 393 248 156 93 49
1839 1657 1475 1318 1169 873 621 442 300 174 91
2077 1263 813 556 426 294 175 102 61 34 20
2400 2020 1692 1464 1271 922 654 458 316 199 120
821 418 292 190 136 88 57 35 26 17 8
876 700 551 459 392 288 200 143 83 50 34
7 5 4 4 2 0 0 0 0 0 0
5 4 3 2 0 0 0 0 0 0 0
B
K-M Survival curves(CSS)
LND:T
LND=No, T=T1
LND=No, T=T2
LND=No, T=T3
LND=No, T=T4
LND=Yes, T=T1
LND=Yes, T=T2
LND=Yes, T=T3
LND=Yes, T=T4
T1
T2
T3
T4
Survival probability
100%
75%
50%
25%
0%
Log-rank
p < 0.0001
0 12 24 36 48 60 72 84 96 108 120
Time (month)
Number at risk
LND:T
2215 1739 1370 1081 898 621 393 248 156 93 49
1839 1657 1475 1318 1169 873 621 442 300 174 91
2077 1263 813 556 426 294 175 102 61 34 20
2400 2020 1692 1464 1271 922 654 458 316 199 120
821 418 292 190 136 88 57 35 26 17 8
876 700 551 459 392 288 200 143 83 50 34
7 5 4 4 2 0 0 0 0 0 0
5 4 3 2 0 0 0 0 0 0 0

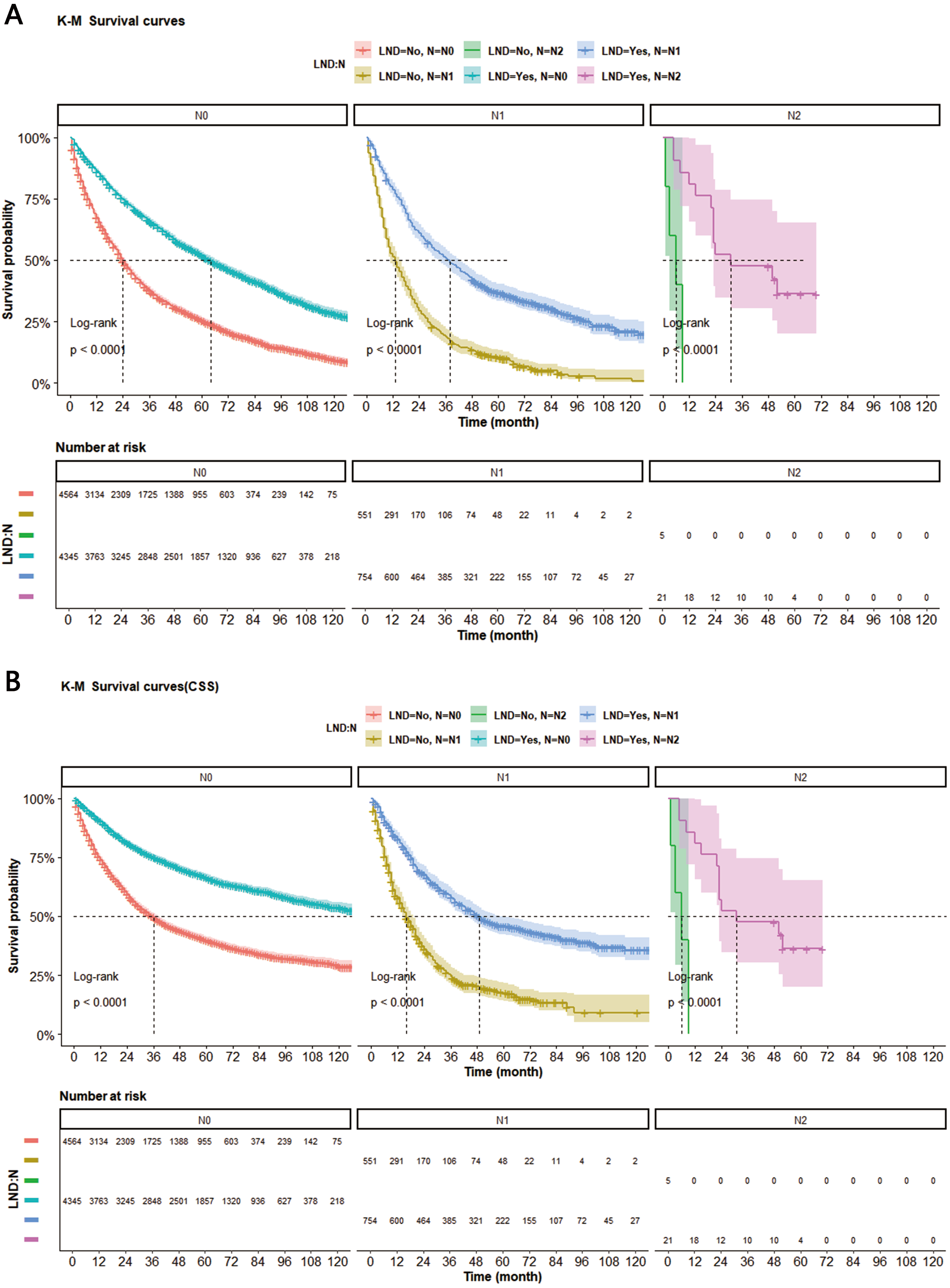
A
K-M Survival curves
LND:N
LND=No, N=N0
LND=No, N=N1
LND=No, N=N2
LND=Yes, N=N0
LND=Yes, N=N1
LND=Yes, N=N2
N0
N1
N2
Survival probability
100%
75%
50%
25%
0%
Log-rank
p < 0.0001
0 12 24 36 48 60 72 84 96 108 120
Time (month)
Number at risk
LND:N
4564 3134 2309 1725 1388 955 603 374 239 142 75
551 291 170 106 74 48 22 11 4 2 2
5 0 0 0 0 0 0 0 0 0 0
4345 3763 3245 2848 2501 1857 1320 936 627 378 218
754 600 464 385 321 222 155 107 72 45 27
21 18 12 10 10 4 0 0 0 0 0
B
K-M Survival curves(CSS)
LND:N
LND=No, N=N0
LND=No, N=N1
LND=No, N=N2
LND=Yes, N=N0
LND=Yes, N=N1
LND=Yes, N=N2
N0
N1
N2
Survival probability
100%
75%
50%
25%
0%
Log-rank
p < 0.0001
0 12 24 36 48 60 72 84 96 108 120
Time (month)
Number at risk
LND:N
4564 3134 2309 1725 1388 955 603 374 239 142 75
551 291 170 106 74 48 22 11 4 2 2
5 0 0 0 0 0 0 0 0 0 0
4345 3763 3245 2848 2501 1857 1320 936 627 378 218
754 600 464 385 321 222 155 107 72 45 27
21 18 12 10 10 4 0 0 0 0 0

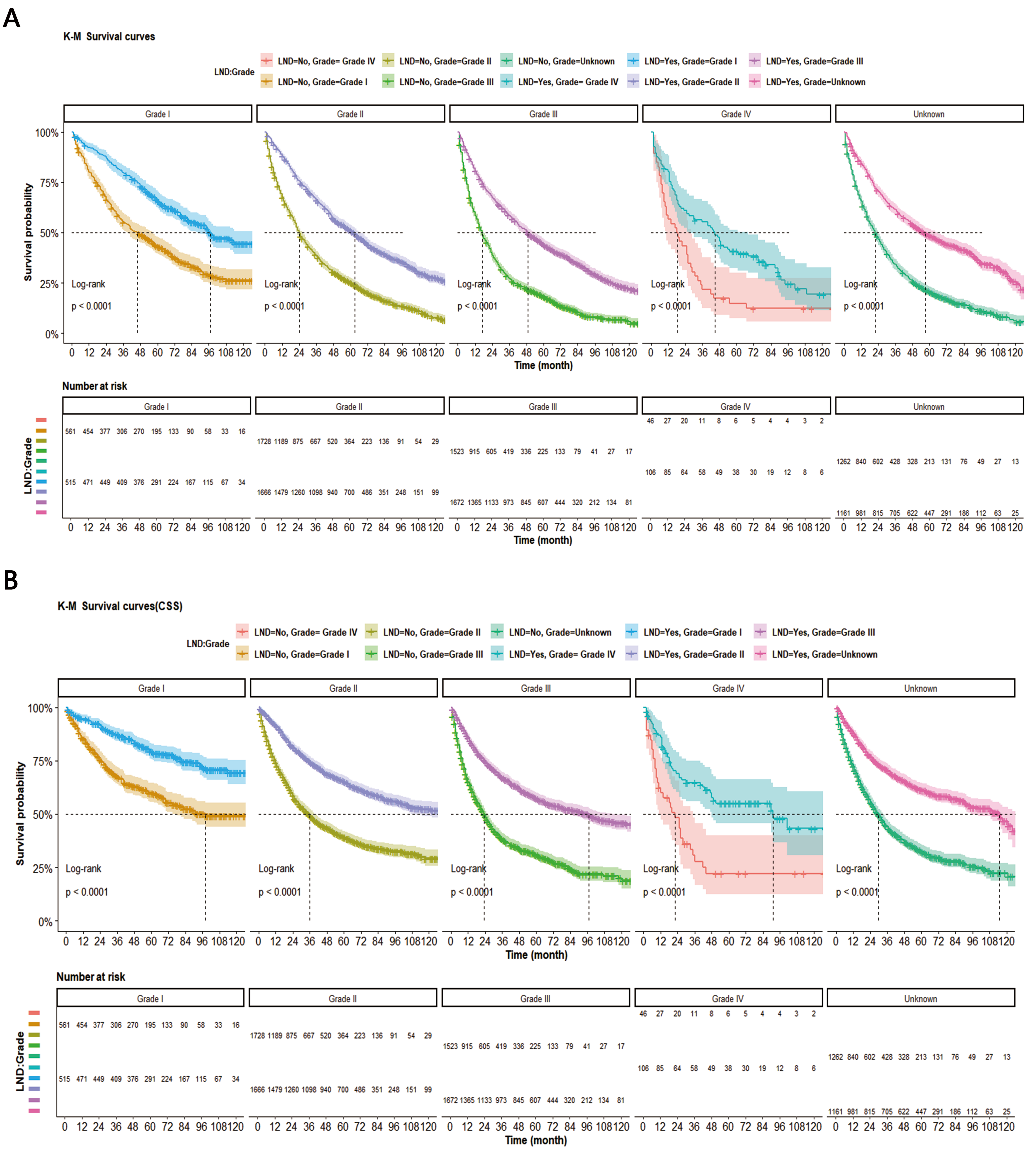
A
K-M Survival curves
LND:Grade
LND=No, Grade= Grade IV
LND=No, Grade=Grade I
LND=No, Grade=Grade II
LND=No, Grade=Grade III
LND=No, Grade=Unknown
LND=Yes, Grade= Grade IV
LND=Yes, Grade=Grade I
LND=Yes, Grade=Grade II
LND=Yes, Grade=Grade III
LND=Yes, Grade=Unknown
Grade I
Grade II
Grade III
Grade IV
Unknown
Survival probability
Log-rank
p < 0.0001
Time (month)
Number at risk
LND:Grade
B
K-M Survival curves(CSS)
Survival probability
Log-rank
p < 0.0001
Time (month)
Number at risk
LND:Grade

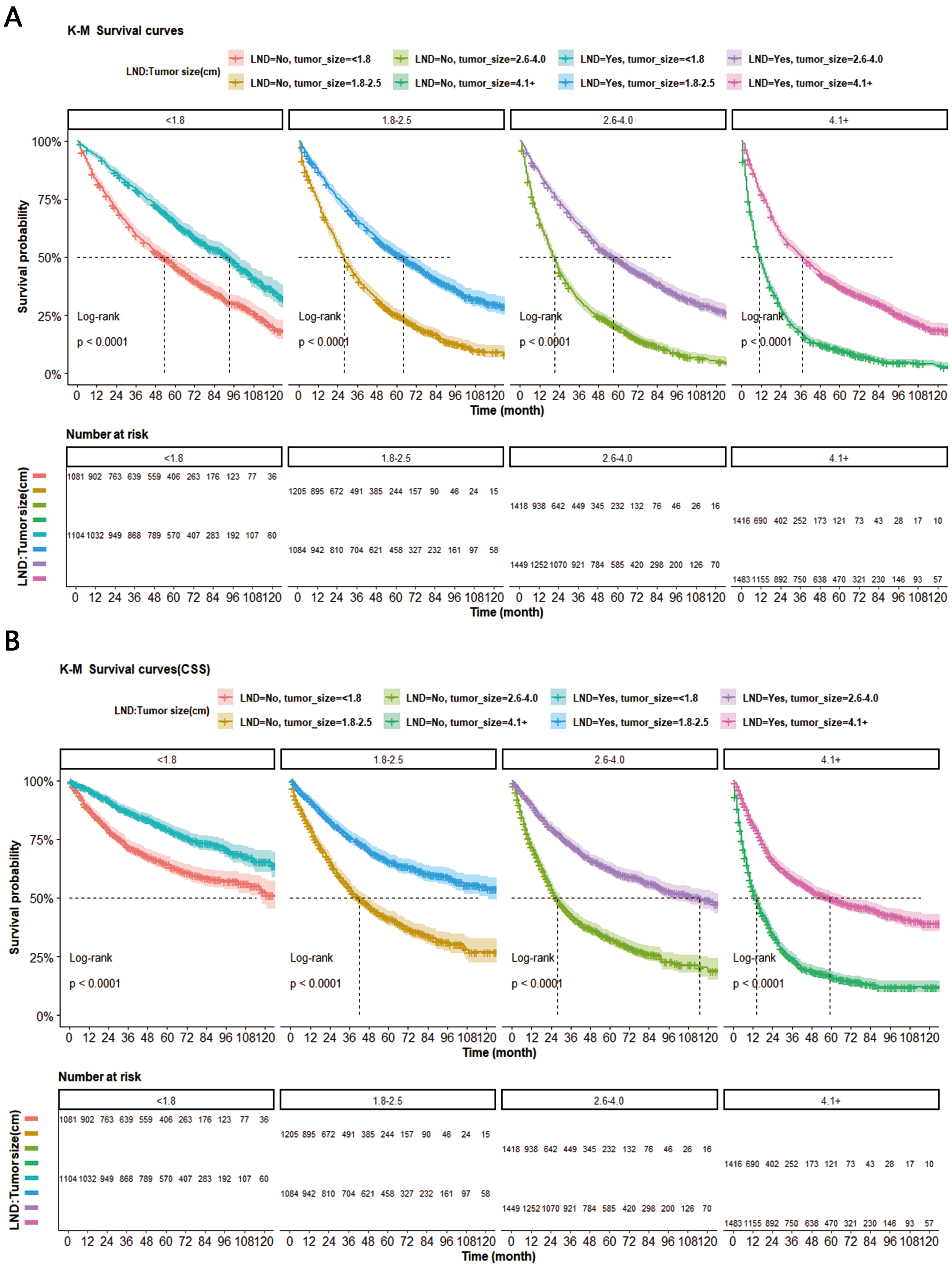
A
K-M Survival curves
LND:Tumor size(cm)
LND=No, tumor_size=<1.8
LND=No, tumor_size=1.8-2.5
LND=No, tumor_size=2.6-4.0
LND=No, tumor_size=4.1+
LND=Yes, tumor_size=<1.8
LND=Yes, tumor_size=1.8-2.5
LND=Yes, tumor_size=2.6-4.0
LND=Yes, tumor_size=4.1+
<1.8
1.8-2.5
2.6-4.0
4.1+
Survival probability
100%
75%
50%
25%
0%
Log-rank
p < 0.0001
0 12 24 36 48 60 72 84 96 108 120
Time (month)
Number at risk
LND:Tumor size(cm)
1081 902 763 639 559 406 263 176 123 77 36
1104 1032 949 868 789 570 407 283 192 107 60
1205 895 672 491 385 244 157 90 46 24 15
1084 942 810 704 621 458 327 232 161 97 58
1418 938 642 449 345 232 132 76 46 26 16
1449 1252 1070 921 784 585 420 298 200 126 70
1416 690 402 252 173 121 73 43 28 17 10
1483 1155 892 750 638 470 321 230 146 93 57
Time (month)
B
K-M Survival curves(CSS)
LND:Tumor size(cm)
LND=No, tumor_size=<1.8
LND=No, tumor_size=1.8-2.5
LND=No, tumor_size=2.6-4.0
LND=No, tumor_size=4.1+
LND=Yes, tumor_size=<1.8
LND=Yes, tumor_size=1.8-2.5
LND=Yes, tumor_size=2.6-4.0
LND=Yes, tumor_size=4.1+
<1.8
1.8-2.5
2.6-4.0
4.1+
Survival probability
100%
75%
50%
25%
0%
Log-rank
p < 0.0001
0 12 24 36 48 60 72 84 96 108 120
Time (month)
Number at risk
LND:Tumor size(cm)
1081 902 763 639 559 406 263 176 123 77 36
1104 1032 949 868 789 570 407 283 192 107 60
1205 895 672 491 385 244 157 90 46 24 15
1084 942 810 704 621 458 327 232 161 97 58
1418 938 642 449 345 232 132 76 46 26 16
1449 1252 1070 921 784 585 420 298 200 126 70
1416 690 402 252 173 121 73 43 28 17 10
1483 1155 892 750 638 470 321 230 146 93 57
Time (month)
